# Metacommunity Composition of Web-Spiders in a Fragmented Neotropical Forest: Relative Importance of Environmental and Spatial Effects

**DOI:** 10.1371/journal.pone.0048099

**Published:** 2012-10-23

**Authors:** Ronei Baldissera, Everton N. L. Rodrigues, Sandra M. Hartz

**Affiliations:** 1 Programa de Pós-Graduação em Ecologia, Departamento de Ecologia, Universidade Federal do Rio Grande do Sul – UFRGS, Porto Alegre, Rio Grande do Sul, Brazil; 2 Programa de Pós-Graduação em Biologia Animal, Departamento de Zoologia, Universidade Federal do Rio Grande do Sul – UFRGS, Porto Alegre, Rio Grande do Sul, Brazil; Utah State University, United States of America

## Abstract

The distribution of beta diversity is shaped by factors linked to environmental and spatial control. The relative importance of both processes in structuring spider metacommunities has not yet been investigated in the Atlantic Forest. The variance explained by purely environmental, spatially structured environmental, and purely spatial components was compared for a metacommunity of web spiders. The study was carried out in 16 patches of Atlantic Forest in southern Brazil. Field work was done in one landscape mosaic representing a slight gradient of urbanization. Environmental variables encompassed plot- and patch-level measurements and a climatic matrix, while principal coordinates of neighbor matrices (PCNMs) acted as spatial variables. A forward selection procedure was carried out to select environmental and spatial variables influencing web-spider beta diversity. Variation partitioning was used to estimate the contribution of pure environmental and pure spatial effects and their shared influence on beta-diversity patterns, and to estimate the relative importance of selected environmental variables. Three environmental variables (bush density, land use in the surroundings of patches, and shape of patches) and two spatial variables were selected by forward selection procedures. Variation partitioning revealed that 15% of the variation of beta diversity was explained by a combination of environmental and PCNM variables. Most of this variation (12%) corresponded to pure environmental and spatially environmental structure. The data indicated that (1) spatial legacy was not important in explaining the web-spider beta diversity; (2) environmental predictors explained a significant portion of the variation in web-spider composition; (3) one-third of environmental variation was due to a spatial structure that jointly explains variation in species distributions. We were able to detect important factors related to matrix management influencing the web-spider beta-diversity patterns, which are probably linked to historical deforestation events.

## Introduction

The historical process of Atlantic Forest deforestation resulted in the present pattern of many forest fragments distributed in a matrix of different land uses [1], creating the possibility for non-directional variation in metacommunity composition [2] among the network of forest remnants. The study of factors driving the variation in species composition among forest patches is of utmost importance in order to properly manage the fragmented and highly disturbed Atlantic Forest. Several recent studies have increased understanding of the causes and consequences of Atlantic Forest loss and fragmentation, showing that variation in arthropod species composition among fragments is affected by human disturbance, and that mosaics of native and managed forests can harbor a significant portion of arthropod diversity [1], [3], [4], [5], [6]. The Atlantic forest is recognized for its large number of species and high number of endemic species [7]. However, this biome is one of the most highly threatened tropical forests, because its deforestation has been closely related to the economic exploitation of different commodities [4]. The remaining Atlantic forest is composed mainly by small isolated fragments composed by second-growth forests in early to medium stages of succession, and much of the remaining forest is subjected to strong matrix influences [1]. Additionally, recently changes to the Brazilian Forestry Code proposed by Brazilian Congress may result in serious harm for the remaining Atlantic forest fragments inserted in private properties, causing the loss of biodiversity and ecosystem services [8].

The study of the variation in community composition among sites – beta diversity [9] – is important to understand many ecological and biogeographical issues, such as the origin and distribution of diversity [10], [11], [12]. It also provides valuable information for conservation-biology questions such as the identification of intersection areas and transition zones, and helps to determine the number and arrangement of protected areas required to characterize the diversity within a region of interest [13], [14], [15]. “If beta diversity is entirely the result of contemporary and historical random processes, resources can be taken anywhere in the region without adverse effects as long as we are not depleting them” [16]. Otherwise, the environmental and spatial features of an environment maintaining beta diversity need to be preserved. Two basic mechanisms have been proposed to explain variations in beta diversity [16]. The environmental-control mechanism proposes that variation in environmental characteristics is responsible for the variation in species composition through the differentiation of available niches, which favors the establishment of diverse assemblages of species. The second mechanism highlights the importance of neutral mechanisms [17], [18]. Thus, beta diversity would emerge through the limitation of species dispersal, which would create aggregated patterns inducing spatial autocorrelations in species distributions.

Spider communities respond to natural and human-caused environmental changes [19], [20]. At local scales, richness and abundance of spiders can be strongly influenced by the vegetation structure [3], [21]. Therefore, spiders may be good indicators of the variation in habitat structure within forest fragments, a factor directly linked to historical human disturbances. Recent studies have shown that spider metacommunities tend to be controlled mainly by environmental or climatic effects, with an increasing influence of spatial variables at broader scales, indicating that these arthropods are not limited by dispersal at small-sized landscapes [22], [23], [24]. However, the relative importance of environmental and spatial factors to the composition of arthropod metacommunities in Neotropical fragments is poorly known. In the context of metacommunity ecology [25], the partitioning of environmental and spatial factors influencing beta diversity may indicate potential causal mechanisms (species interactions/dispersal) for explaining observed patterns of species distributions in the network of local communities [26]. Briefly, metacommunities can be described by four conceptual frameworks [25], [26], [27]: *species-sorting* and *mass-effects* acts both on the assumption of environmentally heterogeneous habitat patches and unlimited dispersal. The difference is that the former assumes species differ in the ability to cope with the environmental heterogeneity, while the latter assumes that dispersal is frequent enough to allow for persistence at sink habitats. The *patch-dynamics* concept assumes that habitat patches are environmentally homogeneous, species differ in their ability to disperse, and there is a colonization-competition trade-off. Finally, in *neutral-model* metacommunities species do not differ in their fitness or niche, i.e they are ecologically equivalent; the distribution of composition is linked to differences in the dispersal potential of species along the geographic spatial extent. Although this classification “is not directly operational because it is difficult to link mechanisms to a single paradigm” [27], it can bring important insights about the metacommunity structure. In the present study, we analyzed the relative influence of environmental and spatial variables on the patterns of variation of web-spider metacommunity composition in fragments of the Atlantic Forest in a landscape mosaic in southern Brazil. We asked the following questions: (1) What is the relative importance of purely environmental, spatially structured environmental, and purely spatial variables on variations in web-spider metacommunity composition? (2) What environmental variables, represented by vegetation structure and patch metrics, best explain the variation of metacommunity composition of web spiders in patches of Atlantic Forest? (3) Is there a combined effect of patch metrics and vegetation structure on web-spider composition, i.e., is there a hierarchically structured effect of both sets of variables on web-spider composition? To our knowledge the first question has not yet been addressed in studies of arthropod fragmentation in the Neotropics. Based on the metacommunity paradigms and on web-spider ecology, our hypotheses were that (1) local predictors, mainly vegetation structure, exerts the greatest influence on web-spider beta diversity, and there is a hierarchical effect of patch metric variables (patch-level) on vegetation structure variables (plot-level) associated with human activities [28], [29]; (2) spatial variables are not significant descriptors of web-spider beta diversity, because these animals display a passive dispersal mode (ballooning) that allows random distribution of colonizers over the landscape [30], [31], [32].

## Methods

### Study Area

The study area is located in the Municipality of Torres, a coastal plain in the State of Rio Grande do Sul, southern Brazil (UTM Coordinates 22 J 65756248 N –620219 E). The altitude in the region ranges from 0 to 90 m.a.s.l., with a subtropical mesothermic and humid climate. The mean annual minimum and maximum temperatures varied from 15.6 to 22.3°C respectively, and total annual precipitation was 1387 mm – meteorological data for 1962–90 [33].

**Figure 1:**
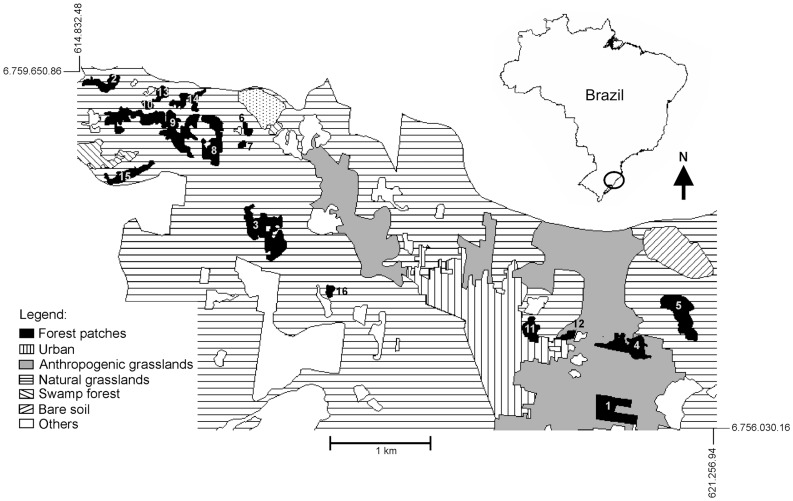
Land-use map of the landscape mosaic. Map showing the 16 forest fragments of Atlantic Forest and the fragment surroundings. Coordinates are in UTM system (22 J).

The site was originally covered with Atlantic Forest vegetation, part of the Tropical and Subtropical Moist Broadleaf Forest biome, Serra do Mar Coastal Forests ecoregion [34]. This is the southern limit of the Atlantic Forest (*stricto sensu*) classified as Dense Ombrophilous Forest [35]. Field work was performed in a landscape mosaic representing a slight gradient of urbanization (Fig. 1) running from west to east, with higher levels of urbanization in the east. The rural matrix consisted of small farms, with cattle breeding and farming as the main activities. Forest fragments were basically second-growth forests with old remnant trees interspersed with different degrees of regenerating stratum. Recently disturbed patches had fewer regenerating trees in the understory. The surrounding patches are of six types: agriculture (mostly beans, corn, and sugarcane), banana plantations, *capoeira* (forest succession early initial stage), *Eucalyptus* plantations, pasture (anthropogenic grassland class of Fig. 1), and buildings (urban class of Fig. 1) Plantations and *capoeira* are inserted in the natural grasslands class of Fig. 1.

### Sampling Design

We sampled 16 forest fragments ranging from 0.4 to 13.7 ha in size (Table 1), distributed in a landscape *ca*. 26 km^2^ in area (Fig. 1). We chose the fragments based on accessibility, landowner permission, and absence of recent grazing activities. A proportional sampling procedure was used to set the number of sampling units in each fragment. The 12 smaller fragments were divided into two sampling units, whereas the medium-sized (2) and large (2) were subdivided in three and four sampling units per fragment, respectively. In total, 38 sampling units were studied. Digitalized maps were inspected prior to fieldwork, to set the coordinates of the centers of each fragment. We used the coordinates in the field to determine the sampling units. In the small fragment, the sampling units were located 28 m inward from the edge. Each sampling unit consisted of plots 12×2×2 m. The plot height was measured from 0.20 m up to 2.20 m above the ground. We selected the sampling units in the field by sorting two, three, or four of the eight basic geographic directions. The plots’ vertices were at least 10 m apart from each other. Sampling units within each fragment were pooled in order to perform the analyses (see the *Scaling beta diversity* section).

**Table 1:** Description of environmental variables and XY coordinates.

	Size (ha)	X	Y	Number of bushes	PCA1	Shape
P1	3.8	5663	1512.10	12	1.954	1.0024
P2	2.4	559.64	4764.66	15	1.564	1.1234
P3	12	2234.07	3330.70	86	1.337	1.1283
P4	6.9	5983.46	2096.93	83	1.845	1.1568
P5	4.9	6432.11	2537.56	9	1.143	1.0759
P6	0.8	2025.77	4316.11	4	0.654	1.0360
P7	0.4	1985.71	4171.91	12	0.654	0.9946
P8	6.3	1689.29	4139.87	52	1.625	1.0902
P9	13.7	1264.67	4404.24	88	1.826	1.2018
P10	0.6	1024.32	4572.49	42	1.136	0.9939
P11	2.8	4917.93	2297.22	44	1.625	1.0229
P12	1.1	5310.50	2241.13	48	1.143	1.0425
P13	1.3	1160.53	4692.66	20	2.046	1.0550
P14	2.4	1489.01	4636.59	71	1.735	1.0420
P15	1.3	816.02	3875.50	21	1.136	1.0691
P16	1.1	2866.98	2681.76	30	0.654	1.0218

PCA1 =  site scores obtained by PCA ordination over a matrix of land-use types surrounding the fragments. Variance explained by axis 1 = 73%.

### Spider Sampling

We collected only web spiders because (1) they are easy to find with the aid of a web highlighter, in our case wheat flour; and (2) the distributions of these animals are strongly linked to variations in vegetation structure [28]. Therefore, web spiders were manually collected by visual inspection of each plot after the collector spread the powder over the vegetation. Collections were made during dry days from March through to July 2009. The time spent in each plot depended on the amount and complexity of its vegetation, so from four to 20 hours were required to sample one individual plot. Each web spider collected was stored in an individual vial in 70% ethanol. Adult web spiders were identified by the second author. Voucher specimens are deposited in the spider collection of the Museu de Ciências Naturais of the Fundação Zoobotânica do Rio Grande do Sul, in Porto Alegre, Brazil.

### Environmental Variables

#### Patch vegetation structure

We measured ten variables in each plot, to characterize the forest-fragment structure: number of trees (woody plants >3 m), number of bushes (woody plants between 0.2 and 3 m), number of palm-trees, number of woody vines, presence of multistrata (0 for two strata, 1 for multistrata), number of tree/bush leaves, number of tree/bush branches, number of tree/bush twigs, number of tree/bush dry branches, and number of vine branches. We counted all individuals of the first five variables within each plot. The last five variables were estimated by a point-counting method [3]: The collector positioned a 2.2 m-long pole vertically along 36 horizontal equidistant points in each plot, and the plant structures touching the pole were counted and classified. The Shannon diversity index (*H′*) was calculated in order to achieve a measurement of understory vegetation diversity, and it was inserted as an additional variable in the environmental vegetation matrix along with the other 10 vegetation variables.

#### Fragment metric variables

We measured five fragment metric variables: log-transformed area, nearest-neighbor distance, patch shape complexity, level of urbanization, and surrounding land use. The first four variables were measured from data in a land-use classification shape file generated in ArcView Gis 3.3 (Environmental Systems Research Institute, Inc.) [36]. Fractal dimension *Frac* = 2l n[(P/4)/ln(A)] was used to assess patch shape, where *P* is the fragment perimeter and *A* is the patch area [37]. Lower values of fractal dimension indicate squared or more-structured fragments (usually man-made), and higher values indicate more complex shapes (natural). From the Fig. 1, we divided the fragments in roughly two groups, depending on the level of urbanization. Fragments with high levels of urbanization were inserted in the east portion of the landscape showing the presence of urban, bare soil, and anthropogenic grasslands land usesFragments in the west portion were within rural areas showing mainly the presence of natural grasslands land use. In order to characterize the land uses surrounding each fragment, we checked *in situ* the types of land uses contiguous to each one. Six types of land uses were found: *capoeira*, *Eucalyptus* plantation, buildings, pasture, agriculture, and banana plantation. A presence-absence matrix of land uses surrounding the 16 fragments was then constructed, and a principal component analysis (PCA) based on a product moment resemblance measure between variables was performed. The first ordination axis was then used as the surrounding land-use variable. The analysis was carried out using the software Multiv v.2.1 [38].

#### Climatic variable

The air temperature measured for each day of collection was computed from the Torres Meteorological Station data (INMET, Brazil, http://www.inmet.gov.br/). The data for each fragment were averaged by the number of days spent to sample the sampling units. Therefore, we were able to account for temporal variation in web-spider composition associated with the temporal range of our sampling (March – July).

### Spatial Variables

We derived the spatial variables by using principal coordinates of neighbor matrices (PCNM), a method well suited for the detection of spatial trends across a wide range of scales [39], [40], [41]. The XY coordinates of the center of each forest fragment were used to construct a Euclidean distance matrix. Then, this matrix was truncated at the smallest distance that keeps all sites connected in a single network, which corresponds to the maximum distance between two fragment centers in one dimension (2,068.68 m in our case). The truncated portion was filled with an arbitrarily large distance value. Then, a principal coordinates analysis (PCoA) was carried out, and the eigenvectors associated with positive eigenvalues were retained as spatial variables (PCNM variables) [39], [40]. PCNM eigenvectors were created using the function “pcnm” in the “vegan” package for the R language (R Development Core Team 2009).

### Data Analysis

#### Scaling beta diversity

Because our main purpose was to analyze beta diversity *among forest fragments*, we assumed higher beta diversity among fragments, and lower beta diversity within fragments. In order to check this assumption, the software Partition 3.0 [42] was used to hierarchically decompose the total amount of diversity (*gamma*) into the components of mean diversity within fragments (*alpha_2_*) and plots (*alpha_1_*) and diversity among fragments (*beta_2_*) and plots (*beta_1_*). The results corroborated the assumptions: observed beta diversity within fragments (*β* = 6.38) was significantly lower than expected by chance (*P*<0.01), while observed beta diversity among fragments (*β* = 42.94) was significantly higher (*P*<0.01). Therefore, pooled composition was used for each patch, i.e., our working sampling units were the fragments.

In the subsequent analyses we used the “vegan” and “packfor” packages of R language v. 2.13.1 (R Development Core Team 2011) to perform all analyses. We applied the Hellinger transformation [43] to the community-abundance data prior to analyses. Hellinger transformation makes the community-composition data containing many zeros suitable for analysis by linear methods such as redundancy analysis (RDA) [44]. Our general null hypothesis was that web-spider beta diversity was not related to environmental or spatial variables.

#### Beta diversity explained by environment and space

**Figure 2:**
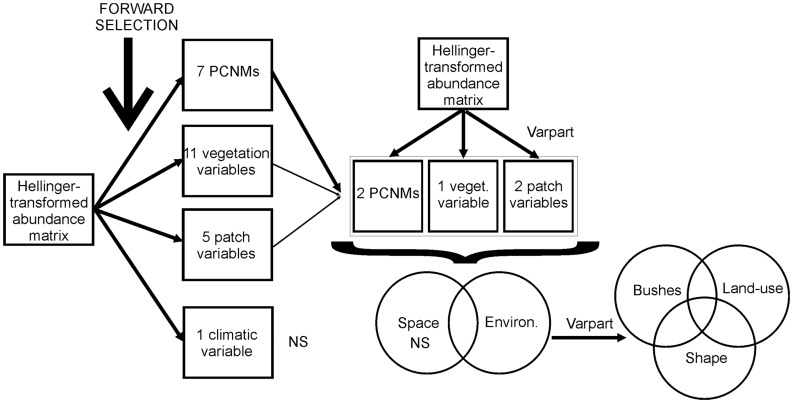
Diagram of the statistical steps. Diagram of the different data sets used and the analytical steps performed with respect to the four predictor data matrices. Varpart  =  variation partitioning. NS  =  not significant. PCNM  =  principal coordinates of neighbor matrices. Analysis initiated by performing a selection of variables from each data matrix. The selected variables were grouped in a unique data matrix, which was subjected to a variation partitioning method to separate the influence of space and environment. Finally, the environmental and spatially structured environmental portion of variation was subjected to the variation partitioning to separate the unique and joint effects of environmental variables on metacommunity structure.

In order to address our first question, we performed two sets of analyses. First, a forward selection analysis based on redundancy analysis (RDA) was run separately for each of the three environmental matrices: vegetation structure, patch metrics, and climate; and for the spatial matrix (Fig. 2). We used the double-stop criterion in the analyses [45]. The procedure began with performing a global test (RDA) with all variables of each data matrix. Afterwards, α-values (*P*<0.1 after 9999 random permutations) and adjusted coefficient of multiple determinations (*R^2^*_adj_) of global tests were used as stopping criteria in the forward selection of variables. The variables that fulfilled both stopping criteria for each matrix were identified as the significant environmental and spatial variables influencing the variation in metacommunity composition.

#### Beta diversity explained by environmental variables

To address our second and third questions, we used the selected environmental variables (vegetation structure + patch metrics) in another variation partitioning to assess the proportions of variation of web-spider composition explained by each environmental variable alone and also by their joint effects (Fig. 2). The joint effects quantify the level of redundancy among variables measured at different scales (within- and between-fragments) [29].

We analyzed the influence of sample size on environmental variables by performing a variance partitioning with the forward selected environmental variables and the number of plots of each fragment as an extra explanatory variable. The results showed that the variance added to the environmental model was negligible (*R^2^*_adj_ <0.0001). Analysis of the independent fraction of the variance of the Hellinger-transformed web-spider abundance matrix explained by the sample size (RDA) was not significant (*pseudo-F* = 0.97, *P* = 0.5).

We realized two tests in order to check for dispersal limitation within the metacommunity. First we regressed the dominance of each local community against the local species richness. If the rates of regional dispersal were high in the metacommunity, we expected an inverse relationship between the two variables [17]. Second, we assessed the decay of community similarity with geographical distance by relating the Sorensen similarity index and the geographical distance calculated between all fragments. A lack of relationship suggests that the metacommunity was not limited by dispersal [32].

## Results

### General Patterns

We collected a total of 3854 web spiders from 16 Atlantic Forest patches. From this total, 55 species from 255 adults were determined and utilized in the analyses. The Chao 1 estimator [46] showed that the number of species collected reached 68% of the absolute number of species in the metacommunity (see web-spider species Checklist S1 for values of alpha diversity).

### Land Use Surrounding the Fragments

The first PCA axis explained 73% of the variation in the composition of land uses surrounding the forest fragments. The values of the correlation coefficients of each variable with PCA axis 1 are presented in Table 2. The lack of a correlation between axis 1 and pasture arose from the fact that all the fragments had pasture in their surroundings. Therefore, we can interpret the PCA axis 1 as a surrounding compositional gradient from solely pasture (more likely to be disturbed by cattle) to more diversified forest-fragment surroundings.

**Table 2:** Values of correlation coefficients between surrounding land-uses of forest fragments qualitatively measured in the field and the first PCA axis based on a product moment resemblance measure between variables.

Surrounding land-uses	R
Buildings	0.77
Farming	0.70
*Capoeira*	0.60
Banana plantation	0.40
*Eucalyptus* plantation	0.39
Pasture	0.00

First axis explained 73% of variation.

### Partition of Environmental and Spatial Effects

Forward selection procedures identified two spatial (PCNM4 [*R^2^*_adj_ = 0.032; *P* = 0.068] and PCNM7 [*R^2^*_adj_ = 0.07; *P* = 0.040]) and three environmental variables (number of bushes [*R^2^*_adj_ = 0.038; *P* = 0.035], land-use surroundings [*R^2^*_adj_ = 0.045; *p* = 0.018] and fragment shape [*R^2^*_adj_ = 0.078; *P* = 0.050]) as significant predictors of web-spider metacommunity variation among forest patches. The climatic matrix did not influence web-spider beta diversity (*pseudo-F* = 1.41; *P* = 0.13). Because the eigenvectors of spatial variables are ordered by decreasing spatial scales [39], we can interpret the PCNM7 variable as representing the fragment scale (grain) and the PCNM4 as an intermediate scale between the fragment and the spatial extent of the study.

The partitioning of environmental and spatial effects showed that the variation attributable to pure environmental effects had a significant influence on web-spider composition, and that the spatial-effect fraction was negligible (Table 3), suggesting that pure neutral processes exert little effect on web-spider composition. The spatial variation shared with the environment (spatially structured environment) explained 4% of the variation.

**Table 3:** Variation partitioning of environmental and spatial effects on web-spider metacommunity composition.

Fractions of variation	R^2^	R^2^_adj_	*F*	*P*
[a+b] Environmental + shared	0.30	0.12	1.68	0.005
[b+c] Spatial + shared	0.19	0.07	1.56	0.01
[a+b+c]	0.43	0.15	1.52	0.005
[a] Only environmental		0.08	1.40	0.025
[b] Environment spatially structured		0.05		
[c] Only spatial		0.02	1.19	0.23
[d] Residual		0.85		

Environmental variables: number of bushes, land use surrounding the patches, shape of patches. Spatial variables: two PCNM variables.

### Influence of Environmental Variables

**Figure 3:**
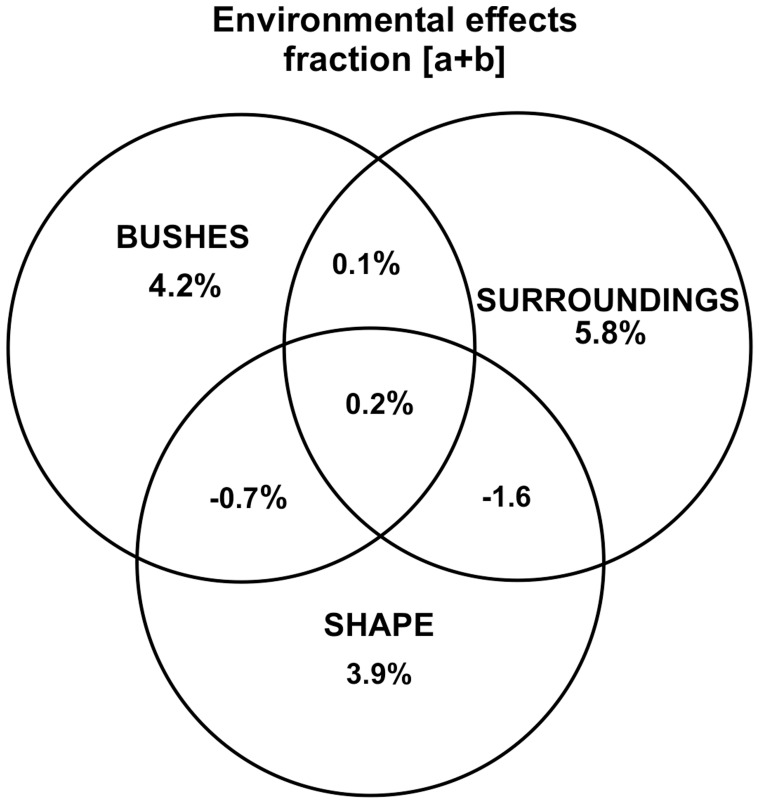
Variation partitioning of environmental variables. Venn diagram showing the results of the variation partitioning procedure carried out on the forward selected environmental variables coupled with a common variation explained by spatial variables (fraction [a+b]).

RDA analysis showed that the three selected environmental variables significantly explained 12% of the web-spider metacommunity variation (Table 3). Of this variation, the land use in the surroundings (49%) was the variable with the highest individual contribution to the variation (Fig. 3). The combined fractions of variation explained by the environmental variables at the plot and patch levels were negligible. Therefore, we did not find hierarchical effects of patch-level variables on vegetation variables.

### Dispersal Limitation

The relationship between local community dominance and richness was significantly negative (R^2^ = 0.725; *P*<0.001). There was no significant relationship between the Sorensen similarity indexes and the geographical distances (R^2^ = 0.003; *P* = 0.539). From these results, we infer that the web-spider metacommunity is not limited by dispersal in the studied landscape.

## Discussion

We detected important features of the variation in web-spider composition among Atlantic Forest fragments. First, spatial legacy [47] was not important in explaining the variation of web-spider composition among fragments. Second, environmental predictors explained a significant part of the variation of web-spider composition. Third, one-third of environmental variation was due to spatial structure – spatial nuisance [47], which together explains variation in species distributions. Additionally, web-spiders seemed to be not limited by dispersal in the studied landscape.

The findings suggested that the web-spiders metacommunity is structured by a species-sorting dynamic at local scale [26]. In that sense, the metacommunity dynamics act by the assortment of different species to complementary niches along the resource gradients [25] represented by the three selected environmental variables. Because we did not find joint effects of predictors on web-spider composition along the gradients, we may infer that there are three vectors of variation influencing the composition. The first is linked to within-fragment amount of vegetation, which is probably a result of the level of human disturbance in the interior of fragments. Second, a compositional gradient is determined by the composition of surrounding land-uses, with the fragments embedded in a more diversified matrix presenting a particular set of species. The third vector is linked to the shapes of fragments and shows changes in the composition as the fragments become more irregular, i.e., less disturbed.

### Absence of Spatial Legacy

The lack of a strictly spatially structured variation in web-spider composition showed that the relative contribution of spatial autocorrelation to the overall pattern was small, a result previously suggested for the same ecoregion [48]. The absence of a purely spatial influence may be due to the lack of dispersal limitation [47]. Most neutral models predict that species composition changes across space because species have limited dispersal distances [17], [49]. We found evidence that the web-spider metacommunity dispersal is not limited, because there was a significant inverse relationship between local community dominance and local species richness [17]. Therefore, high rates of dispersal bring more regional diversity to the local communities, since under high dispersal rates, species occur at lower abundances in local communities having higher species richness [17]. Therefore our results suggest that spatial effects on the web-spider species turnover in human-disturbed landscapes seem to be of minor importance when compared to the effects of reduction of suitable habitat in combination with habitat fragmentation [23], [50].

The study extent may also play an important role in determining the initial similarity between plots, with the extent and the initial similarity exhibiting a significant negative relationship [32]. The lack of this negative relationship in our study shows the presence of a lower species turnover in the landscape, therefore relaxing the effect of dispersal limitation on web-spider metacommunity variation. Nevertheless, contrasting results of the importance of spatial effects were found in spider studies performed in other biomes. For example, the relative importance of spatial variables (24.5%) in structuring an alpine spider community was high [51], while a lack of significant correlation between spider assemblage composition and geographic distance was found in a study in Spain carried out over a geographic extent similar to the present study [22]. Broader spatial scales, e.g. along climatic gradients, appear to structure spider metacommunities mainly by a joint effect of environment and space [24], but for butterflies the increase of spatial extent seem to produce a spatially structured metacommunity independent of environmental dissimilarity among local habitats [52]. De Meutter et al. [53] found indication that an aquatic arthropod metacommunity composed of passive dispersers was structured both by an environmental factor and a significant pure spatial contribution; therefore, suggesting that the metacommunity was a species-sorting/mass-effect type.

### Environmental Effects

Environmental variables accounted for most of the explained variation in web-spider metacommunity composition. The amount of vegetation was the only variable linked to forest structure that influenced the composition. None of the microhabitat variables was selected as a significant environmental effect on the web-spider composition, as also found in another Atlantic Forest study [54]. We found a gradient of change in local web-spider communities linked to the change of understory vegetation quantity. This finding highlights the importance of variables linked to vegetation structure for the responses of spider composition in the landscape [55]. An extensive study in Central Europe showed that shading by vegetation was one of the main factors driving differences in spider composition between habitats [19]. In the Brazilian Cerrado, plant density was also related to the variation in spider composition among vegetation patches [56]. In the present study, the presence of more closed understory suggests that less-disturbed patches show particular web-spider compositions. It is expected that the time elapsed since a disturbance influences the similarity of web spiders among forest patches [57], [58]. At the patch level, the shape of fragments and land use in the surroundings influenced the variation in web-spider composition. This suggests that the kind and composition of matrix management may be important factors affecting web-spider metacommunity composition. There was a web-spider community linked to fragments that were more likely to be disturbed by cattle (pasture), and to fragments with more structured shapes. Sampling in heavily grazed fragments was avoided, but two fragments within a farmland showed clear signs of previous grazing. Therefore, there must be a web-spider community linked to the initial development of the understory vegetation after cattle disturbance. It is unlikely that this community comes from the intervening matrix (pasture), since a study performed in one of the fragments studied here showed that the composition of understory-dwelling spiders was completely different from that in the contiguous early-stage area, indicating that forest-spider species avoid matrix habitats [59]. This characteristic, coupled with the absence of dispersal limitation, suggests that the local web-spider communities in the early-disturbed patches are composed by individuals dispersing from other forest fragments. This particular set of species would be able to colonize recovering habitats with a low density of understory vegetation. Other studies also highlighted that species with different habitat affinities respond differently to human-generated disturbance [60], [61]. On the other hand, another study did not find effects of the structure of the surrounding matrix on the composition of spiders dwelling in farmlands in Germany [62].

Part of the variation in web-spider composition was spatially structured (fraction [b]). We believe that most of this fraction of variation represents missing predictors that are themselves spatially structured [47]. This is surprising when we consider the range of scales treated in our study; we expected to find more environmental influence.

### Unpredictable Variation

Large amounts of variation in web-spider metacommunity composition remained unexplained. Nevertheless, the proportion of total variance accounted for by environmental variables in our study is similar to those found in three urban areas of Switzerland [23]. These authors suggested that there is a gradient of influence of stochasticity from natural (less stochastic) to urban areas (more stochastic). On the other hand, other variables that were not measured may in fact be important for variations in web-spider composition. In this highly disturbed landscape, human activities such as cattle grazing, extractivism, recreation, and crop diversification may play an important role in web-spider distribution and occurrence. However, we were able to detect important factors related to matrix management influencing the web-spider beta-diversity patterns, which are probably linked to historical deforestation events. This is important when we consider that remnants of the Atlantic Forest are subject to intense human impacts.

### Conservation and Management of Fragments

Little is known about the effects of fragmentation and anthropogenic land-use on the arthropods of Atlantic forest. The results of this study, coupled with a previous one in the same region, suggest that the web-spider metacommunity shows a particular composition of forest-dwelling species, which probably avoid the most disturbed matrix areas. This finding is important because the web-spider composition differentiated the most disturbed from the least disturbed fragments [6]. The intensive use of fragment interiors can damage the vegetation structure and compromise the maintenance of animal natural populations [63]. Assuming a species-sorting paradigm, which is based on the ability of species to cope with different local environmental conditions by means of niche diversification and differences in resource exploitation, we suggest that the intensive use of fragments may prevent their use by forest-dwelling spiders, while the presence of a dense shrub layer may favor their occurrence due to a high resource availability. Additionally, intensive use of fragment surroundings, mainly as pasture, may prevent the arrival of forest species to local fragments. Therefore, more diversified surroundings may enhance the connectivity in the landscape to dispersing individuals of web-spiders acting as corridors, and providing a high apportionment of potential forest-dwelling colonizers. However, identifying the effects of disturbances on the diversity of a particular locality or region is only the first step toward the conservation of Atlantic Forest fragments.

## Supporting Information

Checklist S1 **List of web-spider species.** List of the web-spider species found in 16 fragments of Atlantic Forest in southern Brazil. Data are ordered by decreasing abundance.
(PDF)
