# Supplementary material for: Metacommunity Composition of Web-Spiders in a Fragmented Neotropical Forest: Relative Importance of Environmental and Spatial Effects
Source: PLoS One. 2012 Oct 23;7(10):e48099. doi: 10.1371/journal.pone.0048099 (PMC3479130; doi:10.1371/journal.pone.0048099)
Supplement: Checklist S1 — List of web-spider species. List of the web-spider species found in 16 fragments of Atlantic Forest in southern Brazil. Data are ordered by decreasing abundance. (PDF) [file pone.0048099.s001.pdf]

Species	Family	P1	P2	P3	P4	P5	P6	P7	P8	P9	P10	P11	P12	P13	P14	P15	P16	Total
Achaearanea passiva	Theridiidae	0	1	6	1	1	3	0	1	2	1	0	5	1	4	0	1	27
Chrysometa itaimba	Tetragnathidae	2	1	2	2	1	0	0	6	3	2	3	0	2	1	0	1	26
Philoponella fasciata	Uloboridae	1	1	1	0	7	1	0	0	0	0	0	0	2	0	6	0	19
Tidarrem haemorrhoidale	Theridiidae	1	0	0	0	1	1	3	1	0	1	0	0	3	1	0	6	18
Sphecozone personata	Linyphiidae	0	0	1	2	0	1	0	0	1	0	0	10	1	0	1	0	17
Theridion quadripartitum	Theridiidae	0	0	1	0	1	0	0	9	0	0	0	0	1	0	0	4	16
Alpaida canoa	Araneidae	0	0	0	2	0	0	0	2	2	2	0	0	0	0	3	0	11
Faiditus striatus	Theridiidae	0	3	2	2	1	1	1	0	0	0	0	0	0	0	0	1	11
Alpaida alticeps	Araneidae	0	0	0	0	0	0	0	0	0	6	0	0	0	0	0	3	9
Theridion calcynatum	Theridiidae	0	0	1	0	0	1	5	0	0	0	0	0	0	0	0	2	9
Hahnia sp.	Hahniidae	1	0	1	2	1	0	0	1	0	0	2	0	0	0	0	0	8
Thymoites sp.	Theridiidae	0	0	0	0	0	0	0	0	0	0	1	3	1	1	1	0	7
Cyclosa fililineata	Araneidae	0	1	1	3	0	0	0	0	0	0	0	0	0	0	0	0	5
Leucauge sp.3	Tetragnathidae	1	0	2	0	1	1	0	0	0	0	0	0	0	0	0	0	5
Achaearanea tesselata	Theridiidae	0	0	0	0	0	0	1	0	0	0	0	1	0	1	0	0	3
Cyclosa morretes	Araneidae	0	0	0	0	1	1	1	0	0	0	0	0	0	0	0	0	3
Echinotheridion sp.	Theridiidae	0	0	0	0	0	0	0	0	1	0	0	0	0	2	0	0	3
Leucauge sp.1	Tetragnathidae	0	1	0	0	0	0	2	0	0	0	0	0	0	0	0	0	3
Neospintharus rioensis	Theridiidae	0	0	0	0	0	0	0	0	1	1	0	0	0	0	0	1	3
Phoroncidia reimoseri	Theridiidae	2	0	0	0	0	0	0	0	0	0	0	1	0	0	0	0	3
Theridion olaup	Theridiidae	0	0	0	0	0	0	0	0	0	0	0	0	0	0	0	3	3
Wagneriana iguape	Araneidae	0	0	0	3	0	0	0	0	0	0	0	0	0	0	0	0	3
Araneus unanimus	Araneidae	0	0	0	0	0	0	1	0	0	0	0	0	0	0	0	1	2
Ariamnes longíssimus	Theridiidae	0	0	0	0	0	0	0	0	1	0	0	0	0	1	0	0	2
Dolichognatha sp.	Tetragnathidae	0	1	0	0	0	0	0	0	0	0	0	0	1	0	0	0	2
Exalbidion sp.	Theridiidae	0	0	0	0	0	0	0	0	0	0	0	1	0	1	0	0	2
Faiditus affinis	Theridiidae	1	0	0	0	0	0	0	0	0	0	0	0	1	0	0	0	2
Nephila clavipes	Nephilidae	0	1	1	0	0	0	0	0	0	0	0	0	0	0	0	0	2
Philoponella sp.	Uloboridae	0	0	0	0	0	0	0	0	0	1	0	0	0	0	1	0	2
Tekellina sp.	Theridiidae	0	0	0	0	0	0	0	0	0	0	0	0	1	1	0	0	2
Wamba crispulus	Theridiidae	0	0	0	1	0	0	0	0	0	0	0	0	0	1	0	0	2
Wendilgarda sp.	Theridiosomatidae	0	0	0	0	0	0	0	1	0	0	0	0	1	0	0	0	2

Achaearanea rioensis	Theridiidae	0	0	0	0	0	1	0	0	0	0	0	0	0	0	0	0	1
Achaearanea taim	Theridiidae	0	0	0	0	0	0	0	0	1	0	0	0	0	0	0	0	1
Anodoration claviferum	Linyphiidae	0	0	0	0	0	0	0	0	0	0	0	0	1	0	0	0	1
Chrysso sp.	Theridiidae	1	0	0	0	0	0	0	0	0	0	0	0	0	0	0	0	1
Cyclosa inca	Araneidae	0	0	0	0	0	0	0	0	1	0	0	0	0	0	0	0	1
Dipoena atlantica	Theridiidae	0	0	0	0	0	0	0	0	0	0	1	0	0	0	0	0	1
Dipoena santacatarinae	Theridiidae	0	0	0	1	0	0	0	0	0	0	0	0	0	0	0	0	1
Dipoena sp.	Theridiidae	0	1	0	0	0	0	0	0	0	0	0	0	0	0	0	0	1
Dipoena variabilis	Theridiidae	0	0	0	0	0	0	0	0	0	1	0	0	0	0	0	0	1
Eustala sp.	Araneidae	0	0	0	0	0	0	1	0	0	0	0	0	0	0	0	0	1
Faiditus sicki	Theridiidae	0	0	0	0	0	0	0	0	0	0	0	1	0	0	0	0	1
Larinia montecarlo	Araneidae	0	1	0	0	0	0	0	0	0	0	0	0	0	0	0	0	1
Leucauge sp.2	Tetragnathidae	0	0	0	0	0	0	1	0	0	0	0	0	0	0	0	0	1
Miagrammopes sp.	Uloboridae	0	0	0	0	0	0	0	0	0	0	0	0	0	0	1	0	1
Mysmenidae sp.	Mysmenidae	0	1	0	0	0	0	0	0	0	0	0	0	0	0	0	0	1
Ocrepeira gnomo	Araneidae	0	0	0	1	0	0	0	0	0	0	0	0	0	0	0	0	1
Rhomphaea brasiliensis	Theridiidae	0	0	0	0	0	0	0	0	0	1	0	0	0	0	0	0	1
Scoloderus cordatus	Araneidae	0	0	0	0	0	0	0	1	0	0	0	0	0	0	0	0	1
Sphecozone diversicolor	Linyphiidae	0	0	0	0	0	1	0	0	0	0	0	0	0	0	0	0	1
Spintharus gracilis	Theridiidae	0	0	0	0	0	0	0	0	0	0	0	0	1	0	0	0	1
Theridion teresae	Theridiidae	0	0	1	0	0	0	0	0	0	0	0	0	0	0	0	0	1
Thwaitesia affinis	Theridiidae	0	0	0	0	0	0	0	0	1	0	0	0	0	0	0	0	1
Wirada sigillata	Theridiidae	0	0	0	0	0	0	0	0	0	0	0	1	0	0	0	0	1
**Total abundance (N)**		**10**	**13**	**20**	**20**	**15**	**12**	**16**	**22**	**14**	**16**	**7**	**23**	**17**	**14**	**13**	**23**	**255**
**Total richness (S)**		**8**	**11**	**12**	**11**	**9**	**10**	**9**	**8**	**10**	**9**	**4**	**8**	**13**	**10**	**6**	**10**	
